# Clinical Outcomes of Mitral Valve Repair in Dogs With Pulmonary Hypertension Secondary to Myxomatous Mitral Valve Disease

**DOI:** 10.1111/jvim.70106

**Published:** 2025-04-22

**Authors:** Tomohiko Yoshida, Darcy Adin, Katsuhiro Matsuura

**Affiliations:** ^1^ Department of Clinical Veterinary Medicine Obihiro University of Agriculture and Veterinary Medicine Obihiro Japan; ^2^ Department of Small Animal Clinical Sciences College of Veterinary Medicine University of Florida Gainesville Florida USA

**Keywords:** canine, cardiac surgery, echocardiography, left‐sided heart disease, prognosis

## Abstract

**Background:**

Myxomatous mitral valve disease (MMVD) can cause pulmonary hypertension (PH). Mitral valve repair (MVR) has been described as a surgical treatment option for MMVD, but the benefit of MVR when PH is present is unknown.

**Hypothesis/Objectives:**

To investigate the change in echocardiographic variables and long‐term outcomes of dogs with PH secondary to MMVD after MVR.

**Animals:**

Twenty‐one dogs with PH secondary to MMVD that underwent MVR.

**Methods:**

Inclusion criteria were MMVD dogs that had a high probability of PH according to the metrics established in the American College of Veterinary Internal Medicine (ACVIM) consensus statement on PH in dogs, and that had two follow‐up evaluations. Echocardiographic variables before MVR were compared with the follow‐up evaluations after surgery.

**Results:**

Before surgery, 12 dogs were Stage C whereas 9 dogs were Stage D. Echocardiographically normalized left ventricular internal diameter in diastole (LVIDDN), mitral E wave velocity, the ratio of the left atrial dimension to the aortic annulus dimension (LA/Ao) and tricuspid regurgitation velocity were significantly decreased after surgery (*p* < 0.01). Complete resolution of preoperative clinical signs was achieved in 71.4% of dogs after surgery. However, two dogs had residual PH (9.5%) and three dogs that had resolution of PH post‐operatively had recurrent PH (14.2%).

**Conclusions and Clinical Importance:**

We showed that most dogs with PH before MVR had decreases in estimated pulmonary arterial pressure after surgery as well as resolution of clinical signs. Some dogs had residual PH and late PH recurrence after MVR, which could suggest underlying pulmonary arterial pathology.

**Abbreviations**
ACVIM: American College of Veterinary Internal Medicine
AT/ET: the ratio of acceleration time to ejection time
BSA: body surface area
E': early diastolic wave signal as measured by Tissue Doppler imaging
E velocity: early diastolic mitral inflow velocity
FS: fractional shortening
LA/Ao: the ratio of the left atrial dimension to the aortic annulus dimension
Lat: mitral annulus at the left ventricular lateral wall
LVIDd: left ventricular internal dimension in diastole
LVIDDN: normalized left ventricular internal dimension in diastole
MMVD: myxomatous mitral valve disease
MPA/Ao: main pulmonary artery/aorta
MVR: mitral valve repair
PH: pulmonary hypertension
RAA index: right atrial area index
RVEDA index: right ventricular end‐diastolic area index
RVOT: right ventricular outflow tract
Sep: mitral annulus at the septal wall
TR: tricuspid regurgitation
VHS: vertebral heart score

## 1 Introduction

Pulmonary hypertension (PH) has many different causes in dogs. According to the PH guidelines of the American College of Veterinary Internal Medicine (ACVIM) [1], PH resulting from left heart disease is defined as group 2 PH and is classified into 2 types of causes depending on its mechanism [1]. One type is postcapillary PH, which is caused by passive pressure propagation because of an increase in left atrial pressure [1, 2, 3]. The other is combined pre‐and postcapillary PH, which is associated with progressive disease characterized by marked pulmonary vasoconstriction and vascular remodeling in addition to an increase in left atrial pressure [1, 2, 3]. Differentiating postcapillary PH from combined pre‐and postcapillary PH is thought to be important for prognosis [4, 5].

The most common cause of increased left heart pressure in dogs is myxomatous mitral valve disease (MMVD) [6, 7]. The presence of severe PH worsens the outcome in dogs with MMVD [8]. In human medicine, mitral valve repair (MVR) can improve postcapillary PH caused by left heart failure [9, 10, 11, 12, 13]. It is possible that MVR could also be effective as a treatment for dogs with PH secondary to MMVD. However, to our knowledge, no studies to date have reported outcomes and follow‐up of dogs undergoing MVR with PH secondary to MMVD at the time of surgery. We investigated the changes in echocardiographic variables and long‐term outcome of dogs with MMVD and presumptive group 2 PH that underwent MVR.

## 2 Materials and Methods

### 2.1 Animals

Data were retrospectively obtained from the medical records of dogs that underwent MVR for treatment of ACVIM Stage C or D MMVD at a private clinic between March 2019 and March 2023 [14]. Ethical approval was not required because of the retrospective nature of the study. Data obtained from the case records included history, signalment (age, breed, sex), body weight, owner‐reported clinical signs, physical examination findings, concurrent diseases, previous medical history, hematology, biochemistry, radiographic vertebral heart score (VHS), echocardiographic variables, total anesthesia time, cardiopulmonary bypass time, aorta cross‐clamp time, perioperative complications, and post‐operative complications as well as echocardiographic data collected at each follow‐up visit.

Dogs were included if they met the criteria for high probability of PH (presumptive group 2 PH) as specified by ACVIM consensus guidelines regarding PH [1]. Mitral valve repair surgery was performed under cardiopulmonary bypass in all dogs within 1 month of the diagnosis of PH and MMVD.

### 2.2 Study Protocol

Retrospectively collected data were obtained from dogs with MMVD and presumptive group 2 PH before MVR, and at two follow‐up evaluations after MVR (3 months post‐MVR and at the last visit). The last visit was defined as the 2nd follow‐up evaluation 6–24 months after surgery. Doppler echocardiography and thoracic radiography were used to assess the resolution of congestive heart failure after MVR. Improvement in clinical signs and body weight were evaluated 3 months after surgery. Residual PH was defined as echocardiographically diagnosed high probability of PH after MVR during hospitalization or at 3 months post‐MVR. Recurrent PH was defined as echocardiographically diagnosed high probability of PH at the last visit but not at the 3 months post‐MVR visit. Survival time was counted from the day of surgery to either the day of death (confirmed by medical record review or phone call to owner) or the time of the last visit. The cause of death was ascertained by medical record review for dogs that did not survive to the end of the study period. Dogs still alive at the end of the study were right‐censored. Survival of dogs still alive was confirmed by phone call to the owner at the end of the study.

### 2.3 Echocardiography

Echocardiography was performed using an Aplio 300 with a 5 MHz sector probe (Canon medical system, Tokyo, Japan). Combined conventional echocardiography protocols, including 2‐dimensional, M‐mode, Doppler blood flow, and tissue Doppler imaging techniques from the right and left parasternal long‐ and short‐axis views were used to evaluate the dogs [15, 16]. The following echocardiographic variables were measured: left ventricular internal dimension in diastole (LVIDd); the ratio of the left atrial dimension to the aortic annulus dimension in end‐systole (LA/Ao); left ventricular fractional shortening (FS); early diastolic mitral inflow (E) velocity; the ratio of peak velocity of early diastolic transmitral flow to peak velocity of late diastolic transmitral flow (E:A); early diastolic (E'), and late diastolic (A') wave signals as measured by tissue Doppler imaging at septum (sep) and left ventricular lateral (lat) wall. Normalized left ventricular internal dimension in diastole (LVIDDN) was calculated by an established allometric formula using LVIDd and body weight [17]. Tricuspid regurgitation (TR) velocity was obtained by continuous wave spectral Doppler interrogation of the tricuspid valve from multiple views. The TR velocity maximum was obtained from a dense TR flow profile and defined as the highest velocity obtained without measuring the feathered edge of the signal. The ratio of the main pulmonary artery to the aorta (MPA/Ao) was measured from the standard right parasternal short‐axis view [18]. The right ventricular outflow tract (RVOT) flow was assessed with pulse‐wave Doppler and obtained by placing the sample volume (2 mm) centrally before the pulmonary valve leaflets. The ratio of acceleration time to ejection time (AT/ET) was measured from pulmonary artery flow signals, where AT was measured as the time between the onset of the Doppler flow signal to the peak flow velocity and ET was measured from the onset of the Doppler RVOT signal to the end of the signal. [19] In the left apical 4‐chamber view, the right atrial area (RAA) at the end of ventricular systole and right ventricular end‐diastolic area (RVEDA) were measured according to previous reports and indexed to body surface area (BSA) to evaluate right heart size [20, 21]. The BSA was calculated using the following equation: BSA = 0.101 × body weight^2/3^.

### 2.4 Mitral Valve Repair

Mitral valve repair was performed under cardiopulmonary bypass as previously reported [22, 23]. In brief, the heart was approached via a left intercostal thoracotomy and pericardiotomy. To perform the cardiopulmonary bypass, heparin 400 U/kg (Heparin Sodium Injection; Nipro, Japan) was administered IV. After the induction of cardioplegia, annuloplasty and repair of the diseased mitral apparatus were performed by artificial chordae replacement using Gore‐Tex sutures (CV‐6; WL Gore & Associates Inc., Flagstaff, AZ, USA). After closing the heart, a spontaneous heartbeat occurred after terminal warm reperfusion. Modified ultrafiltration was conducted at the end of the bypass followed by protamine sulfate 4 mg/kg (Protamine Sulfate Injection; Mochida Pharmaceutical Co. Ltd., Japan) administration to antagonize heparin. Rivaroxaban 1.0 mg/kg PO q24h (Xarelto tablets, Bayer Yakuhin Ltd., Osaka, Japan) was prescribed to prevent thrombosis from the day after surgery to 3 months after surgery.

### 2.5 Statistical Analysis

Statistical analyzes were performed using GraphPad Prism 10 (GraphPad Software, San Diego, California, USA). The distribution of the data was evaluated for normality using the Kolmogorov–Smirnov test. Continuous data are shown as medians and ranges. For comparison of variables at each time (pre MVR, 3 months post‐MVR and last visit), a repeated one‐way ANOVA with Bonferroni's post hoc test was used for normally distributed variables, whereas the Kruskal‐Wallis with Dunn's post hoc test was used for variables that were determined to be non‐normally distributed. Categorical data were compared using Fisher's exact test. For survival analysis, a Kaplan–Meier survival curve was generated to determine all‐cause mortality in this population of dogs. The level of significance was set to *p* < 0.05.

## 3 Results

### 3.1 Study Population

Ninety‐five dogs underwent MVR surgery because of MMVD during the study period. Of these, 21 dogs met inclusion criteria, with echocardiographically determined high probability of PH before surgery. The median age was 10.7 (range, 7.1–14.0) years. Ten dogs were castrated males, five were intact males, five were spayed females, and one was an intact female. The median body weight of the dogs was 4.0 kg (range, 2.4–11.3 kg). Breeds included Chihuahua (*n* = 6), crossbreed (*n* = 5), Toy poodle (*n* = 2), Cavalier King Charles spaniel (*n* = 2), Maltese (*n* = 1), Shiba inu (*n* = 1), Pomeranian (*n* = 1), Miniature schnauzer (*n* = 1), Shih tzu (*n* = 1), and Miniature dachshund (*n* = 1). Twelve dogs were ACVIM stage C (57.1%) and nine (42.9%) were ACVIM stage D. Eight dogs (38%) were observed to have syncope. Thirteen dogs (61.9%) had cough and mild to moderate pulmonary edema at the time of examination, and two dogs (9.5%) had body cavity effusions (one with ascites and the other with both ascites and pleural effusion). The remaining six dogs (28.6%) had a history of congestive heart failure but were clinically stable at the time of examination. Before surgery, the following drugs were prescribed for each dog: furosemide (*n* = 12), torsemide (*n* = 9), pimobendan (*n* = 21), angiotensin‐converting enzyme inhibitor (*n* = 8), spironolactone (*n* = 6), amlodipine (*n* = 5), and sildenafil (*n* = 5). All medications used before surgery eventually were discontinued for all dogs except two, for which pimobendan was continued for 1 month after surgery. Four days after surgery, sildenafil 1.0 mg/kg PO q12h (Viagra 25 mg, Camber Pharmaceuticals Inc., New Jersey, USA) was prescribed for two dogs that were syncopal and had residual PH (Case 1 and 2 in Table S1). Additionally, the same dose of sildenafil was prescribed for three dogs with recurrent PH on days 180, 730, and 780 post‐operatively (Case 3–5 in Table S2).

### 3.2 Surgical Outcome

The median total anesthesia time was 427 (range, 270–615) minutes, median cardiopulmonary bypass time was 165 (range, 115–259) minutes, and the median aorta cross clamp time was 109 (range, 73–166) minutes. The dogs were discharged between 6 and 14 days post‐surgery. The number of dogs affected by complications post‐operatively was 33.3% of cases (7/21), including left atrial thrombus (*n* = 1), dermatitis at the surgical site (*n* = 1), atrial fibrillation (*n* = 1), intrathoracic hemorrhage (*n* = 1), thrombocytopenia (*n* = 1), pancreatitis (*n* = 1) and venous thrombosis in the left forelimb diagnosed by computed tomography (*n* = 1). Left atrial thrombus and venous thrombosis were improved with antithrombotic treatment for 1 month: clopidogrel 2 mg/kg PO q24h (Plavix, Sanofi K.K., Tokyo, Japan) and rivaroxaban 1.0 mg/kg PO q24h (Xarelto, Bayer Yakuhin Ltd., Osaka, Japan). Antibiotics were prescribed for the dermatitis: ampicillin 30 mg/kg IV q8h (ampicillin sodium, FUJIFILM Wako Pure Chemical Corporation, Osaka, Japan) and enrofloxacin 5 mg/kg SC q24h (Baytril, Bayer Yakuhin Ltd., Osaka, Japan). Atrial fibrillation was restored to sinus rhythm by administration of sotalol 2 mg/kg PO q24h (Sotacor 40 mg, Sandoz Group AG, Basel, Switzerland) for 3 days. Intrathoracic hemorrhage and thrombocytopenia were treated by fresh whole blood transfusion 20 mL/kg. Pancreatitis was treated for 10 days with fluid therapy and antiemetic drugs (Maropitant citrate, 2 mg/kg SC., Cerenia, Zoetis Japan, Tokyo, Japan).

### 3.3 Changes in Radiographic and Echocardiographic Data After MVR

Echocardiographic and radiographic data before and after MVR are summarized in Table 1. Compared with preoperative corresponding results, the following were significantly decreased postoperatively at 3 months post‐MVR and the last visit, with a significant difference between the two follow‐up visits: HR, VHS, LVIDDN, LA/Ao, and E/E' sep (*p* < 0.05). Although E velocity, FS, and TR velocity were significantly decreased at 3 months post‐MVR and last visit compared with the preoperative echocardiogram, no significant differences were found between the two follow‐up visits. The E/E' lat, RAA index, and RVEDA index did not differ significantly between the preoperative and 3 months post‐MVR; however, they were significantly decreased at the last visit. No significant differences were identified between preoperative and postoperative MPA/Ao or AT/ET values.

**TABLE 1:** Changes in radiographic and echocardiographic variables before MVR and at two follow‐up evaluations (3 months post‐MVR and last visit) in all dogs with PH secondary to MMVD after MVR. The last visit was defined as the evaluation between approximately 6 and 24 months after MVR.

Values	Baseline	3 months	Last visit	*P*
HR (bpm)	168 (108–189)	130 (99–168)*	125 (101–150)*^†^	*^†^< 0.01
VHS (v)	11.8 (9.9–12.6)	10 0.1 (8.8–11.6)*	9.9 (8.6–11.5)*^†^	*^†^< 0.01
LVIDDN	2.26 (1.99–2.62)	1.67 (1.25–2.1)*	1.51 (1.25–1.75)*^†^	*^†^< 0.01
LVIDd (mm)	34.3 (25–45.1)	26.3 (18.7–34.4)	24.3 (18.2–32.3)*^†^	*^†^< 0.01
LA/Ao	2.34 (2.1–3.15)	1.67 (1.34–2.1)*	1.44 (1.22–1.8)*^†^	*^†^< 0.01
FS (%)	55 (35–63)	30.9 (21–45.6)*	35 (24.5–58.2)*	*< 0.01
E velocity (cm/s)	133 (117–190)	73 (53–101)*	78.5 (54.1–96.1)*	*< 0.01
E/E' lat (cm/s)	16.1 (13.5–32.6)	12.24 (6.7–26.9)	9.83 (7.3–13.8)*^†^	*< 0.01, ^†^0.016
E/E' sep (cm/s)	15.2 (10.6–28.4)	11.4 (10.6–21.2)*	9.6 (7.3–12.7)*^†^	*^†^< 0.01
RVOT velocity (cm/s)	98 (79–112)	91 (77–101)	88 (88–105)	0.23
AT/ET	0.38 (0.22–0.52)	0.41 (0.29–0.45)	0.41 (0.26–0.57)	0.55
MPA/Ao	1.03 (0.87–1.23)	1.02 (0.97–1.21)	1.00 (0.88–1.21)	0.84
TR velocity max (m/s)	3.65 (3.4–5.0)	3.0 (2.4–3.67)*	3.2 (2.26–4.2)*	*< 0.01
RAA (cm^2^)	2.36 (0.95–6.63)	2.25 (1.2–7.45)	2.21 (1.1–7.5) *^†^	*0.04, ^†^0.02
RAA index (cm^2^/m^2^)	8.8 (5.1–16.6)	9.6 (6.0–16.8)	8.2 (5.3–16.3)*^†^	*0.02, ^†^0.01
RVEDA (cm^2^)	2.9 (1.7–9.1)	3.1 (1.8–8.8)	2.9 (1.45–8.33)*^†^	*^†^< 0.01
RVEDA index (cm^2^/m^2^)	12.2 (7.9–18.2)	11.9 (9.1–18.5)	9.7 (6.7–17.9)*^†^	*0.02, ^†^ < 0.01

*Note:* Data are expressed as median and range. Asterisk (*) denotes significant difference compared to baseline (*P* < 0.05). † denotes significant difference between values of 3 months post‐MVR and last visit (*p* < 0.05).

Abbreviations: AT/ET, the ratio of acceleration time to ejection time; E velocity, early diastolic mitral inflow velocity; E', early diastolic wave signal as measured by Tissue Doppler imaging; FS, fractional shortening; LA/:Ao, the ratio of the left atrial dimension to the aortic annulus dimension; lat, mitral annulus at the left ventricular lateral wall; LVIDDN, normalized left ventricular internal dimension in diastole; MMVD, Myxomatous mitral valve disease; PA/AO, main pulmonary artery/aorta; PH, Pulmonary hypertension; RAA index, right atrial area index; RVEDA index; right ventricular end‐diastolic area index; RVOT, right ventricular outflow tract; sep, mitral annulus at the septal wall; TR, tricuspid regurgitation.

### 3.4 Case Outcomes After MVR

Recurrent severe mitral regurgitation was not documented during the study period in any dog. No dogs developed recurrent pulmonary edema during the postoperative follow‐up period. Postoperative median body weight was 4.3 kg (range, 2.6–11.4), which was increased compared to preoperative body weight (*p* < 0.01). Preoperative clinical signs (e.g., cough, syncope, ascites or pleural effusion) were present in all dogs and resolved in 71.4% of cases (15/21) between 1 week and 3 months postoperatively. Although 61.9% of dogs (13/21) were observed to cough before surgery, this percentage decreased to 19% (4/21) postoperatively (*p* < 0.01). Similarly, 38% (8/21) of dogs had syncope preoperatively, which decreased to 9.5% (2/21) postoperatively (*p* < 0.01). Two of 21 dogs (9.5%) that were syncopal with ascites and pleural effusion before MVR had temporary resolution after MVR, but syncope returned during the hospitalization period (within 1 week after surgery). These two dogs were diagnosed with residual PH based on the presence of high TR velocity max (Case1: TR velocity max 3.8 m/s., Case 2: TR velocity max 4.0 m/s), mild interventricular septal flattening, right atrial enlargement, right ventricular eccentric enlargement, and notching of the spectral Doppler profile of the right ventricular outflow tract. Although syncope resolved after sildenafil 1.0 mg/kg PO q12h, scant amounts of ascites and pleural effusion remained. The data for these two dogs are summarized in Table S1.

**FIGURE 1:**
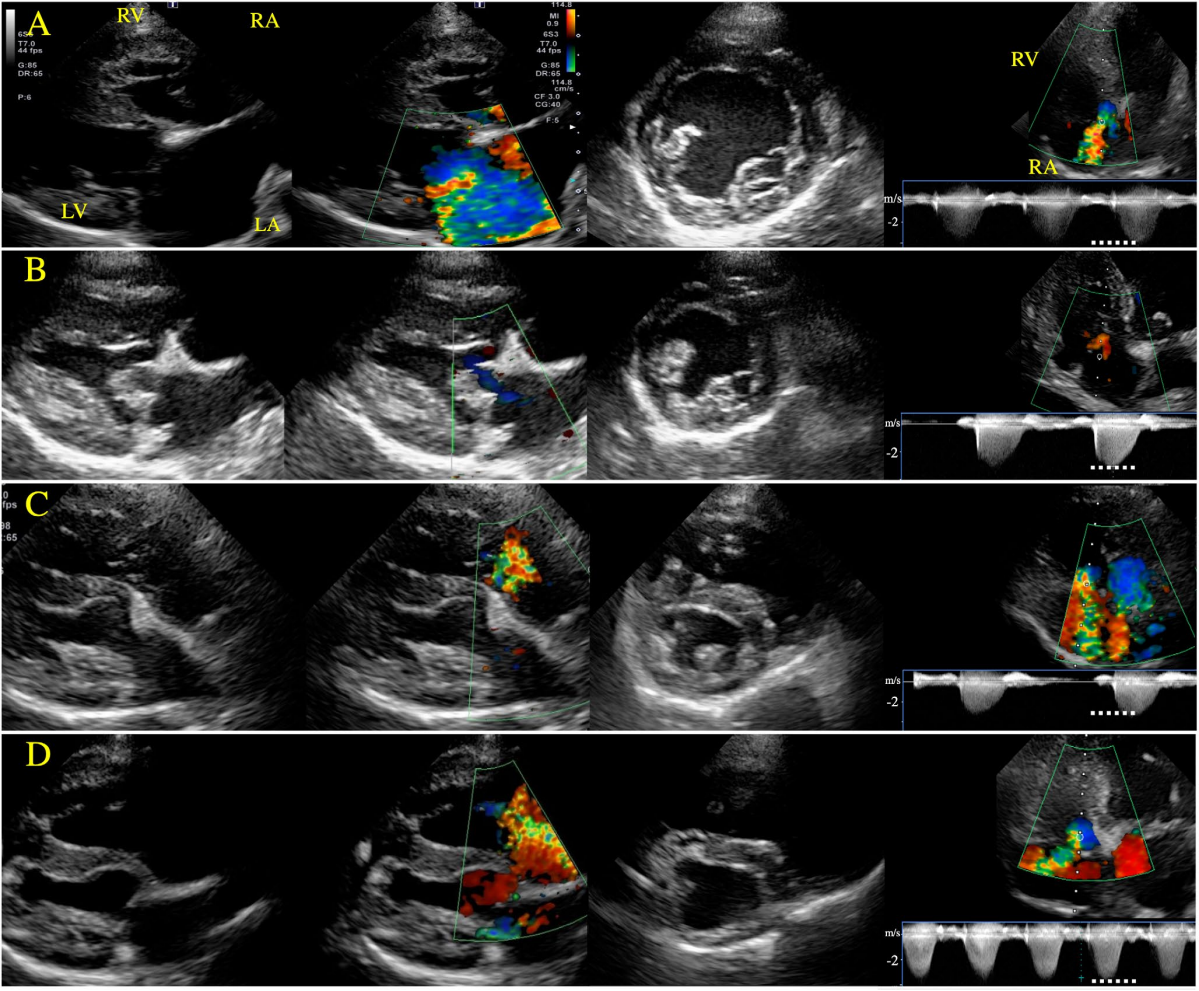
Changes in echocardiographic images in the case 4 dog with recurrent PH before and after mitral valve repair. Two‐dimensional echocardiography images of the right parasternal long axis 4‐chambers view (B‐mode and color Doppler), right parasternal short axis view at the level of the papillary muscles, and left parasternal apical four‐chamber view for measurement of TR velocity max. The solid line shows the alignment TR was measured. (A) Images pre‐MVR. Enlargement of left and right heart was recognized with severe MR and TR (TR velocity max 3.7 m/s). (B) Images at 3 months post‐MVR. The left‐sided heart lumen size and mitral regurgitation flow decreased after surgery. TR velocity max also was decreased (TR velocity max 2.9 m/s). (C) Images at 12 months post‐MVR. Although right heart size was gradually increased, other echocardiographic signs of PH, such as increased TR velocity max and marked flattening of the interventricular septum, were not recognized. (D) Images at 24 months post‐MVR. The dog was diagnosed with PH based on severe eccentric hypertrophy of the right ventricle, underfilling of the left ventricle, right atrial enlargement, flattening of the interventricular septum, and severe TR at increased velocities (TR velocity max 3.45 m/s). Abbreviations: MR, mitral valve regurgitation; MVR, mitral valve repair; PH, Pulmonary hypertension; TR, tricuspid valve regurgitation.

Three other dogs (14.2%) had recurrent PH detected on days 180, 730, and 780 post‐operatively, after showing resolution at the 3 months post‐MVR evaluation. The echocardiographic changes of these three dogs over time are shown in Table S2. Although VHS, LVIDDN, LA/Ao, and E‐wave decreased after MVR, TR velocity, RAA index, and RVEDA index gradually increased after normalizing at the 3 months post‐MVR re‐evaluation. Pulmonary hypertension recurred in dog 3 on day 780 post‐operatively (TR velocity max 4.4 m/s), in dog 4 on day 730 post‐operatively (TR velocity max 3.45 m/s), and in dog 5 on day 180 post‐operatively (TR velocity max 3.7 m/s). These three dogs also had interventricular septal flattening and syncope as a clinical sign of recurrent PH. None of these three dogs were on sildenafil pre‐operatively, but sildenafil 1.0 mg/kg PO q12h was initiated at the time of diagnosis of recurrent PH, and no further syncope occurred. Figure 1 shows changes in echocardiographic images in Dog 4 before and after mitral valve repair. In total, 23.8% (5/21) of dogs had residual PH or recurrent PH after surgery.

**FIGURE 2:**
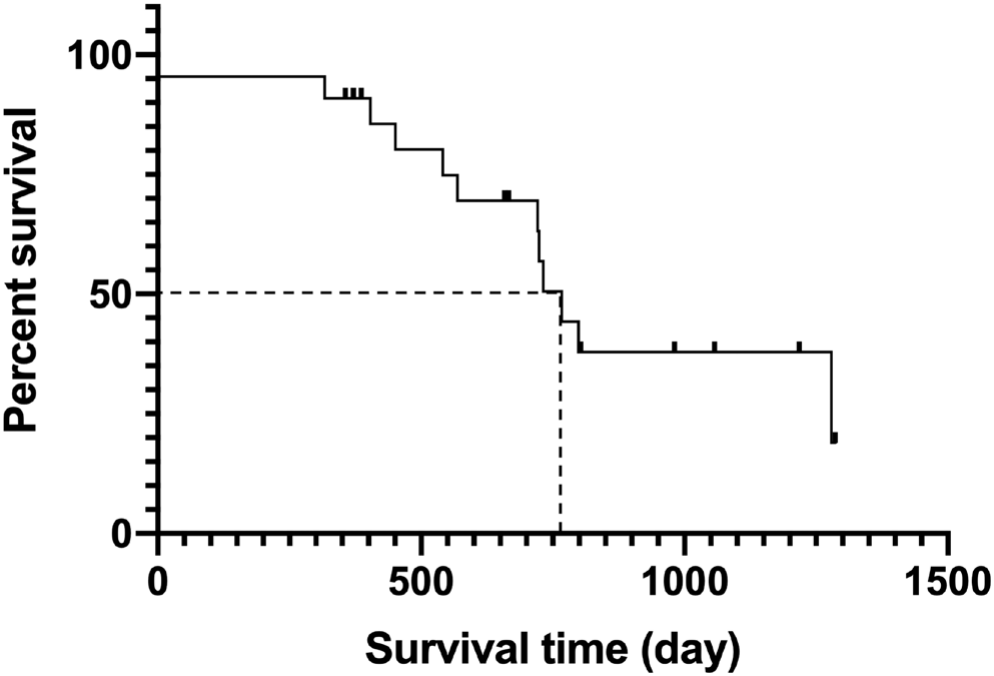
Kaplan–Meier survival curve for 22 dogs with MMVD and PH after MVR. The 50% probabilities of survival are indicated by dotted lines. The median survival time was 767 days (95% CI, 521–851 days). Censored animals are indicated with vertical bars. Abbreviations: MMVD, Myxomatous mitral valve disease; MVR, mitral valve repair; PH, Pulmonary hypertension.

The median survival time (all‐cause mortality) was 767 days (95% confidence interval, 521–851 days) for the 21 dogs in the study (Figure 2). Eleven dogs died during the study period: 5/11 (45.4%) from a malignant tumor, 1/11 (9%) from protein‐losing enteropathy, 1/11 (9%) from portal vein thrombosis, 1/11 (9%) from pancreatitis, 1/11 (9%) from disseminated intravascular coagulation after vaccine allergic shock, and 2/11 (18.2%) from severe PH: The cause of death for the dogs with severe PH was heart failure with ascites and pleural effusion on days 445 and 1279 after surgery. The median follow‐up time for dogs that were alive at the end of the study was 724 days (range, 445–1296 days).

## 4 Discussion

We showed that most dogs with MMVD and PH have a good outcome after MVR. The median survival time of 767 days after surgery in our study compared favorably with the previously reported median survival time of 456 days for dogs with PH secondary to MMVD without surgical intervention [8], suggesting that MVR might improve the prognosis of PH secondary to MMVD. The resolution of PH in most of the dogs in our study after MVR provides evidence that these dogs had group 2 (post‐capillary) PH. This information is useful for screening dogs before MVR by providing information on the likelihood of PH resolution in dogs with both PH and MMVD, and shows that long‐term survival is possible for most dogs with MMVD and PH after MVR.

Despite improved echocardiographic variables of left heart volume loading and clinical signs in most dogs, 23.8% of dogs had residual or recurrent PH after MVR. Therefore, our results also can be used to facilitate discussions with owners about the potential for persistent or recurrent PH that could negatively affect outcomes in some dogs with PH and MMVD even with MVR. The dogs that experienced residual or recurrent PH after MVR (which presumably decreased left atrial pressure based on post‐operative echocardiographic changes) most likely had combined pre‐ and post‐capillary PH. Right heart size and systolic pulmonary artery pressure increased gradually in the dogs with recurrent PH (14.2%).

Our findings align with a previous study that found 17% of people with PH secondary to left heart failure who underwent mitral valve surgery showed progression of PH, which led to increased RV size, worsened RV function, and increased TR [24]. Presumably, some patients with postcapillary PH develop increases in pulmonary artery pressure secondary to long‐standing increased left heart pressures, which can lead to remodeling and contraction of peripheral pulmonary artery vessels that continues even after MVR [24]. Therefore, close monitoring of dogs with progressive increases in right ventricular size and TR velocity is advised because of the potential for recurrent PH. Of the five dogs in our study that presumably had either postcapillary PH or combined pre‐and postcapillary PH, two dogs died from severe PH, and the other three dogs had continuing treatment for progressive PH, suggesting a poor prognosis for this subset of dogs. Future studies are needed to explore ways to identify these dogs because of the adverse effect on clinical outcome. Pre‐operative right heart catheterization might be useful in these patients before MVR.

Our study had some limitations. Because right ventricular function indices were not measured, it is unclear whether there is an association between right ventricular dysfunction and the long‐term prognosis after MVR. In addition, because the actual pulmonary artery pressure and pulmonary artery wedge pressure were not measured with right heart catheterization, detailed hemodynamics from the pulmonary circulation were unknown. The diagnosis of PH was based on echocardiographically estimated peak TR velocity and other supportive findings according to ACVIM guidelines, not invasive pulmonary artery pressure measurement, and this approach could have affected the diagnosis and severity assessment of PH [1]. Additionally, in some dogs, there could have been an error in the measurement of TR velocity because of technical difficulties in obtaining an ideal alignment with eccentric tricuspid regurgitant jets or other factors [25]. Dogs with combined pre‐capillary and post‐capillary PH might benefit from sildenafil; however, the use of phosphodiesterase 5 inhibitors in group 2 pulmonary hypertension dogs warrants caution [1]. Although we ruled out cardiogenic pulmonary edema before using sildenafil in the dogs in our study, the dose administered was higher than that recommended by the ACVIM consensus guidelines [1]. A major limitation of our study is uncertainty about the cause of the PH. These dogs did not undergo complete diagnostic evaluation using computed tomography or magnetic resonance imaging, cytology, catheterization, or intraoperative lung biopsies to screen for diseases contributing to PH. Although, from a clinical point of view, we diagnosed most of these dogs with PH caused by MMVD, this diagnosis was presumptive. However, resolution of PH after MVR for most of the dogs supports this diagnosis. Lastly, the number of dogs with PH caused by MMVD was small.

In conclusion, although the majority of dogs had resolution of PH and good outcomes after MVR, a small proportion had persistent or recurrent PH, suggesting that PH in these dogs was not entirely caused by increased left heart pressures. These findings provide useful information for the preoperative assessment of dogs with MMVD and PH that could aid in decision‐making.

## Disclosure

Authors declare no off‐label use of antimicrobials.

## Ethics Statement

Authors declare no institutional animal care and use committee or other approval was needed. Authors declare human ethics approval was not needed.

## Conflicts of Interest

The authors declare no conflicts of interest.

## Supporting information


**Table S1.** Changes in radiographic and echocardiographic variables before MVR and at two follow‐up evaluations (3 months post‐MVR and last visit) in Case 1 and 2, which had residual PH after MVR.
**Table S2.** Changes in radiographic and echocardiographic variables before MVR and at two follow‐up evaluations (3 months post‐MVR and last visit) in Case 3, 4 and 5 which had recurrent PH after MVR. The last visit was defined as the evaluation between approximately 6 and 24 months after MVR (Case 3 and case 4 last visit: 24 months post‐MVR, Case 5 last visit: 15 months post‐MVR). PH recurred in case 3 after last visit (on day 780 post‐operatively), in case 4 at the last visit (on day 730 post‐operatively), and in case 5 before last visit (on day 180 post‐operatively).
