# Supplementary material for: Clinical Outcomes of Mitral Valve Repair in Dogs With Pulmonary Hypertension Secondary to Myxomatous Mitral Valve Disease
Source: J Vet Intern Med. 2025 Apr 22;39(3):e70106. doi: 10.1111/jvim.70106 (PMC12012645; doi:10.1111/jvim.70106)
Supplement: Supplementary file 1 — Table S1. Changes in radiographic and echocardiographic variables before MVR and at two follow‐up evaluations (3 months post‐MVR and last visit) in Case 1 and 2, which had residual PH after MVR. Table S2. Changes in radiographic and echocardiographic variables before MVR and at two follow‐up evaluations (3 months post‐MVR and last visit) in Case 3, 4 and 5 which had recurrent PH after MVR. The last visit was defined as the evaluation between approximately 6 and 24 months after MVR (Case 3 and case 4 last visit: 24 months post‐MVR, Case 5 last visit: 15 months post‐MVR). PH recurred in case 3 after last visit (on day 780 post‐operatively), in case 4 at the last visit (on day 730 post‐operatively), and in case 5 before last visit (on day 180 post‐operatively). [file JVIM-39-e70106-s001.docx]

| months after surgery (M) | Case 1 Baseline | Case 1 Post  3 M | Case 1  Last  18 M |  | Case 2 Baseline | Case 2 Post  3 M | Case 2 Last  24 M |
| --- | --- | --- | --- | --- | --- | --- | --- |
| HR (bpm) | 157 | 148 | 126 |  | 152 | 142 | 136 |
| VHS (v) | 12.6 | 10.3 | 10.5 |  | 12.2 | 10.8 | 10.8 |
| LVIDDN | 2.62 | 2 | 1.8 |  | 2.23 | 1.67 | 1.49 |
| LVIDd (mm) | 41.8 | 31.6 | 29.0 |  | 42.8 | 32 | 28.7 |
| LA/Ao | 2.48 | 1.43 | 1.31 |  | 2.1 | 1.87 | 1.47 |
| FS (%) | 58 | 38.1 | 35 |  | 59.5 | 36.2 | 26.9 |
| E velocity (cm/s) | 144 | 67.5 | 64.3 |  | 166 | 89 | 86 |
| E/E' lat (cm/s) | 16.8 | 13.7 | 10.3 |  | 16.3 | 10.1 | 3.2 |
| E/E' sep (cm/s) | 12.6 | 12.3 | 9.74 |  | 17.1 | 12 | 5.12 |
| RVOT velocity (cm/s) | 105 | 88 | 95 |  | 99 | 85 | 96 |
| AT/ET | 0.26 | 0.29 | 0.26 |  | 0.28 | 0.33 | 0.45 |
| MPA/Ao | 1.23 | 1.1 | 1.15 |  | 1.21 | 1.2 | 1.15 |
| TR velocity max (m/s) | 4.8 | 3.67 | 3.42 |  | 4.64 | 3.6 | 3.56 |
| RAA (cm^2^) | 3.56 | 3.66 | 3.44 |  | 7.42 | 7.45 | 7.33 |
| RAA index (cm^2^/m^2^) | 12.2 | 12.8 | 11.5 |  | 16.7 | 16.8 | 16.3 |
| RVEDA (cm^2^) | 5.15 | 5.3 | 4.6 |  | 6.1 | 6.23 | 5.21 |
| RVEDA index, cm^2^/m^2^ | 17.7 | 18.5 | 15.4 |  | 13.7 | 14 | 11.6 |

**Supplemental Table 1:** Changes in radiographic and echocardiographic variables before MVR and at two follow-up evaluations (3 months post-MVR and last visit) in Case 1 and 2, which had residual PH after MVR.

The last visit was defined as the evaluation between approximately 6 and 24 months after MVR (Case 1 last visit: 18 months post-MVR, Case 2 last visit: 24 months post-MVR). The post data (3 months post-MVR and last visit) presents the variables in dogs already receiving sildenafil 1 mg/kg PO q12h.

Abbreviations: AT/ET, the ratio of acceleration time to ejection time; E velocity, early diastolic mitral inflow velocity; E’, early diastolic wave signal as measured by Tissue Doppler imaging; FS, fractional shortening; LA/:Ao, the ratio of the left atrial dimension to the aortic annulus dimension; lat, mitral annulus at the left ventricular lateral wall; LVIDDN, normalized left ventricular internal dimension in diastole; M, month; MVR, Mitral valve repair; MPA/Ao, main pulmonary artery/aorta; PH, Pulmonary hypertension; RAA index, right atrial area index; RVEDA index; right ventricular end-diastolic area index; RVOT, right ventricular outflow tract; sep, mitral annulus at the septal wall; TR, tricuspid regurgitation.

| months after surgery (M) | Case 3 Baseline | Case 3 Post  3 M | Case 3 Last  24 M |  | Case 4 Baseline | Case 4 Post  3 M | Case 4 Last  24 M |  | Case 5 Baseline | Case 5 Post  3 M | Case 5 Last  15 M |
| --- | --- | --- | --- | --- | --- | --- | --- | --- | --- | --- | --- |
| HR (bpm) | 156 | 144 | 134 |  | 169 | 145 | 128 |  | 161 | 120 | 115 |
| VHS (v) | 11.5 | 9.8 | 9.9 |  | 11.2 | 9.5 | 9.1 |  | 11 | 10.1 | 9.4 |
| LVIDDN | 2.27 | 1.54 | 1.25 |  | 2.26 | 1.8 | 1.56 |  | 2.21 | 1.52 | 1.65 |
| LVIDd (mm) | 34.1 | 22.8 | 19.6 |  | 34.4 | 27 | 23.9 |  | 44.7 | 30.7 | 32.3 |
| LA/Ao | 2.09 | 1.34 | 1.31 |  | 1.89 | 1.5 | 1.35 |  | 2.2 | 2 | 1.41 |
| FS (%) | 52.6 | 33.8 | 39.6 |  | 63 | 36 | 36 |  | 49 | 21 | 33.1 |
| E velocity (cm/s) | 110 | 64 | 96.1 |  | 115 | 100 | 66.6 |  | 134 | 53 | 66.8 |
| E/E' lat (cm/s) | 11.7 | 11.4 | 10.9 |  | 11.9 | 14 | 5.4 |  | 17.9 | 6.5 | 7.3 |
| E/E' sep (cm/s) | 14.5 | 8.1 | 11.7 |  | 11.9 | 19.6 | 11.56 |  | 16.14 | 6.7 | 7.3 |
| RVOT velocity (cm/s) | 88 | 98 | 89 |  | 100 | 91 | 87 |  | 101 | 96 | 91 |
| AT/ET | 0.52 | 0.44 | 0.46 |  | 0.41 | 0.53 | 0.44 |  | 0.39 | 0.41 | 0.42 |
| MPA/Ao | 1.1 | 1.1 | 0.98 |  | 0.98 | 1.0 | 1.21 |  | 0.92 | 0.95 | 1.12 |
| TR velocity max (m/s) | 3.6 | 2.8 | 4.4 |  | 3.7 | 2.96 | 3.45 |  | 4.38 | 3.3 | 4.2 |
| RAA (cm^2^) | 1.403 | 1.55 | 2.32 |  | 1.335 | 1.4 | 1.88 |  | 6.63 | 7.33 | 7.5 |
| RAA index (cm^2^/m^2^) | 5.5 | 6.3 | 8.2 |  | 5 | 5.5 | 7 |  | 13.7 | 14.6 | 16.1 |
| RVEDA (cm^2^) | 2.21 | 2.6 | 3.21 |  | 2.23 | 2.12 | 3.56 |  | 9.1 | 8.76 | 8.33 |
| RVEDA index (cm^2^/m^2^) | 8.7 | 10.5 | 11.4 |  | 8.5 | 8.3 | 13.3 |  | 18.2 | 17.5 | 17.9 |

**Supplemental Table 2**: Changes in radiographic and echocardiographic variables before MVR and at two follow-up evaluations (3 months post-MVR and last visit) in Case 3, 4 and 5 which had recurrent PH after MVR. The last visit was defined as the evaluation between approximately 6 and 24 months after MVR (Case 3 and case 4 last visit: 24 months post-MVR, Case 5 last visit: 15 months post-MVR). PH recurred in case 3 after last visit (on day 780 post-operatively), in case 4 at the last visit (on day 730 post-operatively), and in case 5 before last visit (on day 180 post-operatively).

Abbreviations: AT/ET, the ratio of acceleration time to ejection time; E velocity, early diastolic mitral inflow velocity; E’, early diastolic wave signal as measured by Tissue Doppler imaging; FS, fractional shortening; LA/:Ao, the ratio of the left atrial dimension to the aortic annulus dimension; lat, mitral annulus at the left ventricular lateral wall; LVIDDN, normalized left ventricular internal dimension in diastole; M, month; MVR, Mitral valve repair; MPA/Ao, Main pulmonary artery/Aorta; PH, Pulmonary hypertension; RAA index, right atrial area index; RVEDA index; right ventricular end-diastolic area index; RVOT, right ventricular outflow tract; sep, mitral annulus at the septal wall; TR, tricuspid regurgitation.
